# Specific inflammatory stimuli that engage innate immune sensors induce novel CD103 expression profiles in macrophages

**DOI:** 10.3389/fcimb.2025.1618339

**Published:** 2025-06-24

**Authors:** Nasry Zane Bouzeineddine, Sebastien Talbot, Sam Basta, Katrina Gee

**Affiliations:** Department of Biomedical and Molecular Sciences, Queen’s University, Kingston, ON, Canada

**Keywords:** CD103/ITGAE, macrophage, inflammation, tlr, virus, innate immunity

## Abstract

The integrin CD103 is an adhesion molecule that facilitates immune cell retention in epithelial tissues through its interaction with E-cadherin. It is a marker for certain CD8+ T-cell subpopulations and conventional type 1 dendritic cells (cDC1), but its presence on macrophages remains poorly characterized. Macrophage differentiation is influenced by M-CSF and GM-CSF, and we investigated whether macrophages can also express CD103 under inflammatory conditions. We examined baseline CD103 expression in bone marrow-derived macrophages (BMDMs) differentiated in M-CSF or GM-CSF and then stimulated them with pathogen-associated molecular patterns (PAMPs) or examined them following viral infection. We found that CD103 is minimally expressed at baseline but is selectively upregulated in M-CSF-differentiated macrophages after stimulation with endosomal TLR agonists. Mechanistically, p38 MAPK inhibition prevented CD103 upregulation, suggesting that this process is mediated by p38 MAPK signaling. Furthermore, *in vivo* LCMV infection induced CD103 expression on peritoneal macrophages. These findings demonstrate that macrophages can express CD103 under specific inflammatory conditions, challenging the assumption that CD103 is restricted to T cells and dendritic cells. This study expands our understanding of CD103 beyond its recognized roles in T cells and DCs, providing new insight into its regulation by macrophages.

## 1 Introduction

CD103, or integrin αEβ7 (ITGAE), plays a key role in cell adhesion, migration, and lymphocyte homing through its interaction with E-cadherin. By enhancing immune cell retention in epithelial tissues, CD103 contributes to tissue-specific immunity (Xu et al., 2022). It is a well-known marker for migrating and resident T cells and dendritic cells (DCs) in organs such as the skin, gut, lung, spleen, and lymph nodes (del Rio et al., 2010), and has been identified on specific immune cell subsets, including effector memory and regulatory CD8+ T cells, as well as conventional DCs (cDC1) (Uss et al., 2006; Takamura, 2018; Kuhn et al., 2020).

In the lung, CD103+ DCs acquire and process cell-associated viral antigens, migrate to the mediastinal lymph nodes, and cross-present these antigens to CD8+ T cells during influenza A virus infection (Ho et al., 2011). They also regulate the migration, survival, and memory responses of CD8+ T cells (Ng et al., 2018). CD103 also promotes the resolution of lung inflammation, as mice lacking CD103 exhibit increased airway inflammation, tissue damage, and impaired inflammation resolution (Bernatchez et al., 2015). In the intestinal mucosa, CD103+ DCs drive gut-homing T-cell responses and support immune surveillance (Luciani et al., 2022; Sasaki et al., 2022). Although CD103 expression in T cells and DCs is well characterized, its association with macrophages (MΦ) remains poorly defined.

MΦ reside in all tissues throughout the body, recognize and respond to foreign stimuli, and bridge adaptive immune responses through antigen presentation to T cells (Banete et al., 2021; Bouzeineddine et al., 2024). They are highly plastic, adapting their phenotype according to environmental cues (Trus et al., 2020; Petrina et al., 2021). Their differentiation is influenced by cytokines such as macrophage colony stimulating factor (M-CSF) and granulocyte macrophage colony stimulating factor (GM-CSF) (Lacey et al., 2012a; Trus et al., 2020; Petrina et al., 2024). M-CSF is expressed during homeostasis, whereas GM-CSF is typically low but rapidly induced during inflammation, driving a more pro-inflammatory MΦ phenotype (Barilo et al., 2024; Bouzeineddine et al., 2024). Unlike cDC1s and T cells, MΦ usually rely on other integrins (e.g., αMβ2, also known as Mac-1) for adhesion and migration (Cui et al., 2018).

Whether MΦ can upregulate CD103 under specific inflammatory conditions, however, remains unclear. To address this question, we examined CD103 expression in bone marrow-derived macrophages (BMDMs) following exposure to pathogen-associated molecular patterns (PAMPs) or infection with lymphocytic choriomeningitis virus (LCMV). Our results show that CD103 is minimally expressed at baseline but is selectively upregulated in M-CSF-differentiated MΦ after stimulation with endosomal TLR agonists (TLR3, TLR7, and TLR9) and following LCMV infection. Mechanistic studies revealed that p38 MAPK signaling is critical for this process, as its inhibition prevented CD103 induction. We further tested this *in vivo* and observed CD103 expression on peritoneal MΦ (pMΦ) during acute LCMV infection. Overall, these findings indicate that CD103 expression in MΦ is highly regulated and emphasize the need to consider its presence when monitoring immune responses *in vivo*.

## 2 Methods

### 2.1 Mice and reagents

Six-to-eight-week-old C57BL/6 (H-2b) mice were purchased from JAX Laboratories (Bar Harbor, USA) and housed under specific pathogen-free conditions. All procedures were performed according to the guidelines of the Canadian Council on Animal Care and approved by the Queen’s University Animal Care Services. Primary cell cultures were maintained in RPMI (Gibco, Fisher Scientific, Canada) containing 10% fetal calf serum (Gibco, Fisher Scientific, Canada) and 50 μg/mL gentamycin (BioShop, Canada). For BMDM stimulations with TLR ligands, the following were used at 1 μg/mL unless otherwise specified: LPS (TLR4-L) (E. coli O55:B5, Sigma Aldrich, Canada), LTA (TLR2-L) (S. aureus, Cedarlane, Canada), poly(I:C) (TLR3-L) (Cedarlane, Canada), R848 (TLR7-L) (resiquimod, Invitrogen, Canada), and CpG-ODN 1826 (TLR9-L) at 1.5 μM (Invitrogen, Canada). To inhibit intracellular signaling, the p38 MAPK inhibitor SB202190 (10 μM; Sigma Aldrich, Canada) was added to mature BMDMs for 1 hour at 37°C and 5% CO_2 prior to poly(I:C) stimulation. Cell viability was monitored using propidium iodide (PI) (0.5 mg/mL; BioLegend, USA).

### 2.2 Primary cell preparation

BMDMs were generated as previously described (Alatery and Basta, 2008; Alothaimeen et al., 2021). Briefly, bone marrow was flushed from mouse femurs and tibias using 1× PBS, and red blood cells were lysed with 1.66% ammonium chloride for 5 minutes. The remaining cells were then cultured in 6-well tissue culture plates (Corning, USA) supplemented with 25 ng/mL recombinant M-CSF or 4 ng/mL recombinant GM-CSF (Shenandoah Biotechnology, Warwick, PA) (Petrina et al., 2024).These conditions were confirmed by culturing bone marrow cells in 20% supernatant from M-CSF-secreting L929 fibroblasts or GM-CSF-secreting X63Ag8 cells (Siednienko et al., 2011; Misharin et al., 2013). Non-adherent cells were removed, and fresh medium was added on days 3 and 5. After 7 days at 37°C and 5% CO_2_, BMDMs were used for experiments.

Splenocytes were isolated as previously described (Alatery and Basta, 2008; Barilo et al., 2024; Barilo et al., 2025). Briefly, spleens were homogenized and red blood cells were lysed with 1.66% ammonium chloride for 5 minutes. The remaining cells were then utilized for flow cytometry. Lung tissue was isolated as described by Misharin et al (Misharin et al., 2013). Briefly, mouse lungs were perfused with 1× PBS and excised. The lungs were cut into small pieces, placed in lung digestion buffer (RPMI containing 5% FCS, 150 U/mL collagenase I, and 50 U/mL DNase I), and incubated for 1 hour at 37°C with intermittent shaking every 10–15 minutes. The cell suspension was then filtered through a 70 μm cell strainer, treated with 1.66% ammonium chloride to lyse red blood cells, and the remaining cells were used for flow cytometry.

For CD103+ BMDC generation, we followed the protocol by Mayer et al (Alothaimeen et al., 2021). Briefly, 15 × 10^6^ bone marrow cells were cultured in 10 mL RPMI with 10% FCS, 50 μM β-mercaptoethanol (Sigma Aldrich, Canada), 5 μg/mL gentamycin, 200 ng/mL FLT3L (provided by Dr. Talbot, Queen’s University), and 4% supernatant from GM-CSF–secreting X63Ag8 cells. On day 5, 5 mL of fresh medium was added. On day 9, non-adherent cells were harvested, counted, and replated at 3 × 10^6^ cells in 10 mL of fresh medium. Non-adherent cells were collected again on days 15–16. Resident peritoneal cells were obtained by injecting 5–7 mL of cold PBS into the peritoneal cavity (Siednienko et al., 2011). Cells were then collected and used immediately for flow cytometric analysis.

### 2.3 Quantitative PCR

Total RNA was extracted using the PuroSPIN™ Total RNA Purification Kit (Luna Nanotech, Canada). After reverse transcription, cDNA was amplified with AzuraView GreenFast qPCR Blue Mix HR (FroggaBio, Canada) using the following primers (IDT, Canada):

- CD103: 5′-GTCAAGAGCCTGCGTGTGGA-3′ (forward) and 5′-CACCAAGGATCGGCAGTTCAGA-3′ (reverse)
- GAPDH: 5′-GGCATGGACTGTGGTCATGAG-3′ (forward) and 5′-TGCACCACCAACTGCTTAGC-3′ (reverse)

Samples were run on a Bio-Rad CFX96 Real-Time PCR Detection System. Cq values were obtained using CFX Manager Software (Bio-Rad) and analyzed by the ΔCT method. Target gene expression was normalized to the reference gene GAPDH.

### 2.4 Virus propagation and infections

The baby hamster kidney (BHK) fibroblast cell line was used to propagate both LCMV-ARM and LCMV-ARM-GFP, as previously described (Alothaimeen et al., 2020). The LCMV-ARM-GFP strain was originally prepared by Dr. De La Torre at the Scripps Research Institute (La Jolla, USA) and provided to Dr. Watts (University of Toronto, Canada). A FACS-based titration assay was performed to determine viral titre, as described in (Korns Johnson and Homann, 2012). Briefly, BHK cells were infected for 72 hours, and the percentage of GFP-positive infected cells was quantified by flow cytometry. Known-titre controls were used to create a standard curve, and a non-linear curve fit was applied to calculate the plaque-forming units (PFU)/mL.

On day 7 of differentiation in M-CSF or GM-CSF, BMDMs were gently washed twice with 1× PBS, then infected at the indicated multiplicity of infection (MOI) for 1 hour at 37°C and 5% CO_2_. After infection, cells were washed again with 1× PBS and cultured with RPMI containing 10% FCS and 50 μg/mL gentamycin (BioShop, Canada). Cells were harvested at specified time points for further analyses. For *in vivo* infections, mice were injected intraperitoneally with virus in 200 μL of sterile PBS at the indicated PFU doses.

### 2.5 Detection of LCMV-ARM-GFP and LCMV-ARM.

Flow cytometry was used to detect LCMV-ARM-GFP and LCMV-ARM infection. At the desired time points, cells were harvested and washed in 1× PBS. For LCMV-ARM-GFP, GFP fluorescence was measured directly on a CytoFLEX flow cytometer (Beckman Coulter, USA). For LCMV-ARM detection, cells were washed and fixed in 4% paraformaldehyde in PBS for 15 minutes at room temperature, followed by permeabilization with 0.1% saponin in PBS for 15 minutes. Cells were then washed in 0.1% saponin/PBS and incubated for 1 hour at room temperature with rat anti-LCMV-NP (clone VL4) supernatant, as previously described (Mulder et al., 2017). After washing, cells were stained for 30 minutes at room temperature with FITC-conjugated goat anti-rat IgG (Biolegend, USA), acquired on the CytoFLEX flow cytometer, and analyzed using FlowJo (BD, USA).

### 2.6 Flow cytometry analysis

After 7 days of culture in M-CSF or GM-CSF–containing media, BMDMs were harvested for flow cytometry. Cells were washed with FACS buffer (1× PBS, 1% BSA, 0.1% sodium azide) and blocked with anti-mouse CD16/32 (Biolegend, USA) for 15 minutes at 4°C. Cells were then stained with the following antibodies (Biolegend, USA) for 20 minutes at 4°C: anti-CD11b (clone M1/70), anti-CD11c (clone N418), anti-F4/80 (clone BM8), anti-MHC-II (I-A/I-E) (clone M5/114.15.2), anti-CD64 (clone X54-5/7.1), anti-CD24 (clone 30-F1), anti-CD68 (clone FA-11), and anti-CD103 (clone 2E7). Fluorophore panels were:

- Lung & spleen DCs: MHC-II (FITC), CD11c (PE/Cy5), CD11b (APC), CD103 (BV421)
- *In vitro* generated CD103^+^ DCs: MHC-II (FITC), CD11c (PE/Cy5), CD24 (PE), CD103 (BV421)
- BMDMs: CD11b (FITC or PE), CD103 (BV421 or APC)
- Peritoneal MΦ: F4/80 (PE), LCMV-NP (FITC), CD103 (BV421 or APC)

MOPC antibodies served as isotype controls (Biolegend, USA). Samples were acquired on a CytoFLEX flow cytometer (Beckman Coulter, USA) and analyzed using FlowJo software (BD, USA).

### 2.7 Statistical analysis

Statistical significance was determined using one-way or two-way ANOVA with Tukey’s multiple comparisons test (∗p < 0.05, ∗∗p < 0.01, ∗∗∗p < 0.001, ∗∗∗∗p < 0.0001).

## 3 Results

### 3.1 Characterization of CD103+ populations in lung, spleen, and bone marrow-derived dendritic cells

**Figure 1:**
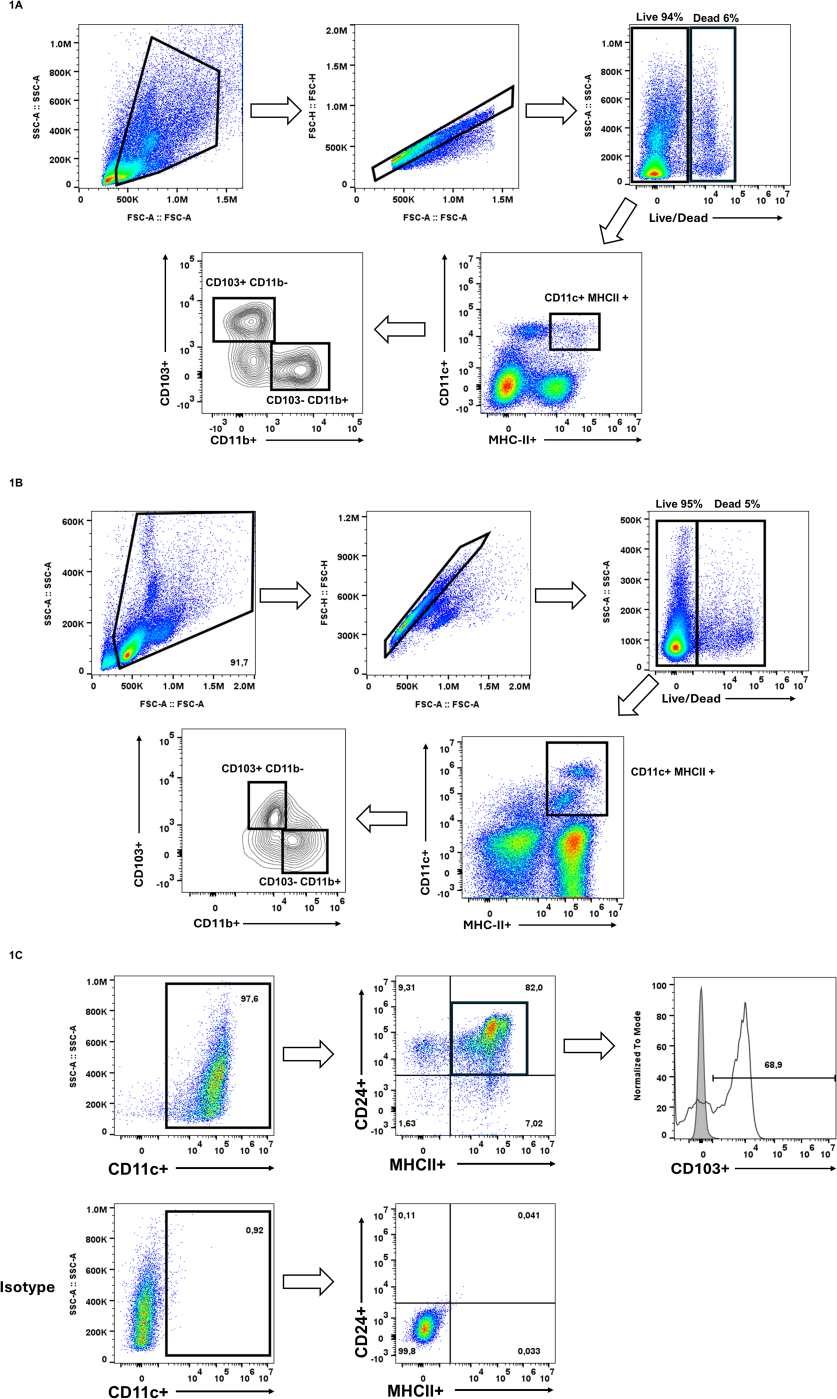
Identification of pre-existing CD103+ populations. **(A)** Single-cell suspensions from digested mouse lung tissue were gated on live (PI^-^), singlet cells, followed by identification of conventional dendritic cells (cDCs) using CD11c and MHCII. Within the CD11c^+^ MHCII^+^ population, two distinct subsets were identified based on the expression of CD103 and CD11b, CD103^+^ CD11b^-^ cDC1 and CD103^-^ CD11b^+^ cDC2. **(B)** Splenocytes were isolated and subsequently gated on live (PI^-^) single cells. cDCs were identified being CD11c^+^ MHCII^+^ cells and subsequently cDC1s were CD103^+^CD11b^-^. **(C)** Bone marrow cells were cultured for 15 days in GM-CSF and FLT3L for the generation of CD103^+^ DCs. Dendritic cells were identified as CD11c^+^. Within this population, cells were further gated on CD24^+^ MHCII^+^, where majority expressed CD103. These results confirm the presence of CD103^+^ populations *in vivo* and *ex vivo*, setting the foundation for investigating whether similar CD103 expression patterns can be found in macrophages also. These results are representative of three independent experiments.

Single-cell suspensions from lung tissue were analyzed by flow cytometry to identify CD103+ populations using sequential gating (**Figure 1A**). Dendritic cells were first gated on CD11c+ and MHCII+. Within this population, cDC1 were identified as CD103+ CD11b-, whereas cDC2 were CD103- CD11b+, consistent with established methods for identifying CD103+ lung-resident DCs (Misharin et al., 2013). We used a similar gating strategy to evaluate spleen-derived CD103+ DCs (**Figure 1B**). Cells were gated on CD11c+MHCII+ and cDC1 were identified as before being CD103+CD11b-, reflecting previous observations of CD103+ DCs in the spleen (Zhan et al., 2011; Backer et al., 2019).

To generate CD103+ DCs *in vitro*, we followed the protocol by Mayer et al. for bone marrow-derived cDC differentiation (Mayer et al., 2014). Cells were cultured with GM-CSF and FLT3L for 15 days, after which they were analyzed by flow cytometry. We gated on CD11c+, then selected CD24+ and MHCII+ cells, finding that 68.9% of the CD11c+ CD24+ MHCII+ population expressed CD103 (**Figure 1C**). These findings confirm that *in vitro* GM-CSF/FLT3L differentiation results in a large population of CD103+ cDC1. Together, these data show that CD103 effectively distinguishes DC subsets across different tissues and can be robustly generated *in vitro*.

### 3.2 CD103 expression is upregulated following PAMP exposure in BMDMs

**Figure 2:**
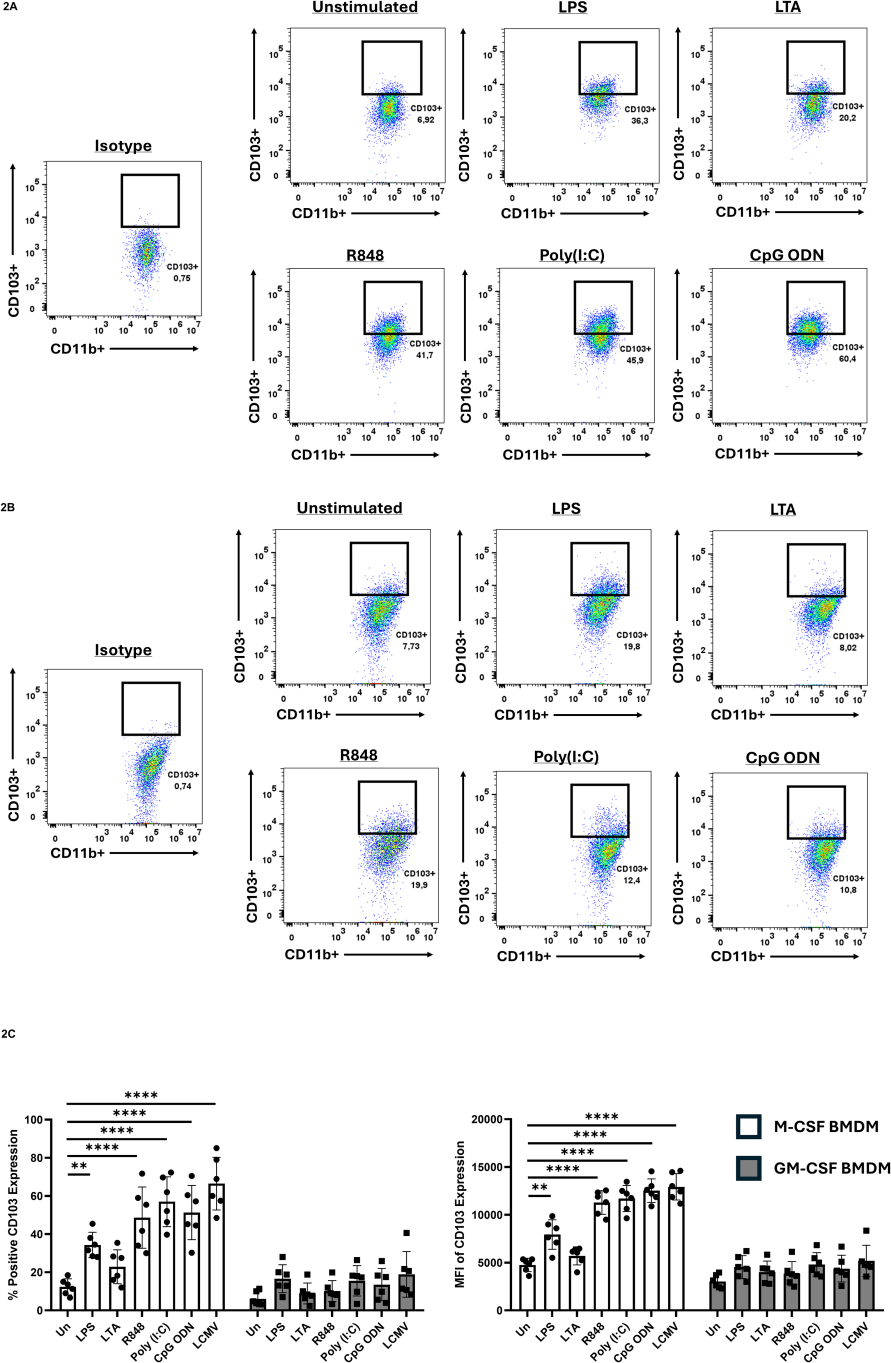
CD103 expression on BMDMs prior/following PAMP exposure. Bone marrow cells were cultured in either M-CSF **(A)** or GM-CSF **(B)** for 7 days, then stimulated with the various TLR ligands for 24h and CD103 expression was evaluated by flow cytometry. Cells were gated on CD11b^+^ populations to identify macrophages. Gates identifying CD11b^+^ CD103^+^ populations were established using isotype controls. **(C)** Quantification of CD103 expression shown by percent positive (left) and mean fluorescent intensity (MFI) (right). CD103 was significantly upregulated in M-CSF differentiated BMDMS following stimulation with specific PAMPS, while GM-CSF differentiated BMDMs had no significant induction. Data are representative of six independent experiments ± SEM. A two-way ANOVA with Tukey’s multiple comparisons test was used to determine statistical significance, **p<0.01, ****p<0.0001.

Previous reports have not described clear CD103 expression in MΦ, particularly in BMDMs. We therefore cultured bone marrow cells for seven days in either M-CSF, which promotes a homeostatic MΦ phenotype, or GM-CSF, which induces a more pro-inflammatory MΦ phenotype (Alothaimeen et al., 2021; Bouzeineddine et al., 2024; Petrina et al., 2024), to assess how these conditions influence CD103 expression. At baseline, CD103 was minimal in both M-CSF- and GM-CSF-differentiated BMDMs, with only a small fraction of cells (~6%) expressing CD103 before PAMP exposure (**Figures 2A, B**). Upon exposure to various PAMPs, including LPS, LTA, poly(I:C), R848, and CpG-ODN, CD103 expression increased selectively in M-CSF-differentiated BMDMs.

In contrast, GM-CSF-differentiated BMDMs did not show a significant CD103 upregulation. Quantification of this response revealed that LPS, R848, poly(I:C), and CpG-ODN all increased CD103 expression in M-CSF-differentiated BMDMs, whereas LTA did not (**Figure 2C**). The strongest upregulation occurred following stimulation with R848, poly(I:C), and CpG-ODN, which engage endosomal TLRs. We next asked whether viral infection that triggers endosomal TLRs could induce a similar response. BMDMs were infected with lymphocytic choriomeningitis virus (LCMV) at a multiplicity of infection (MOI) of 1 for 24 hours. Consistent with TLR7 activation by this negative-sense RNA virus (Alothaimeen et al., 2020), M-CSF-differentiated BMDMs showed pronounced CD103 expression after LCMV infection (**Figure 2C**).

### 3.3 The p38 MAPK signaling pathway drives CD103 upregulation in MΦ

**Figure 3:**
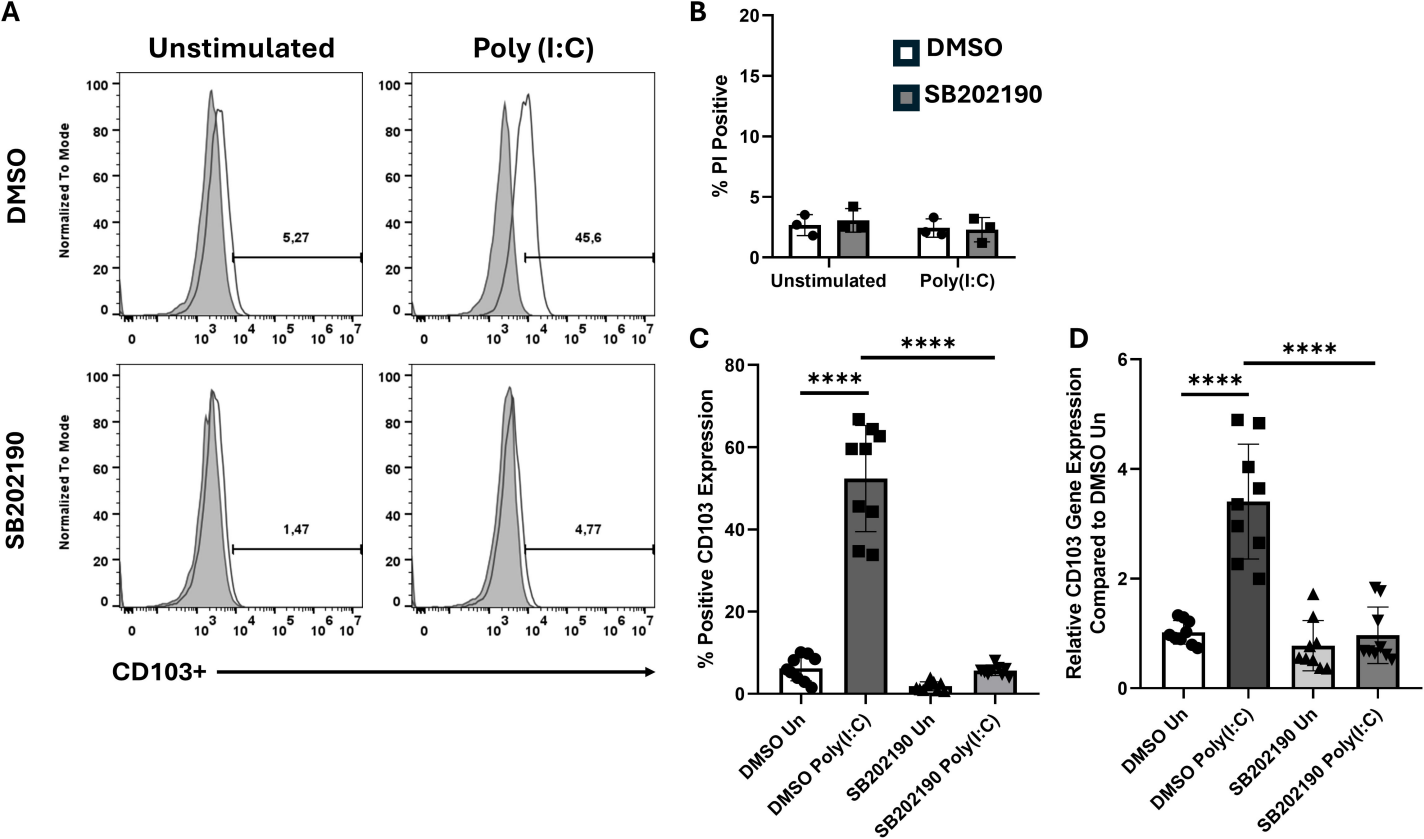
Inhibition of p38 MAPK signaling prevents CD103 upregulation. **(A)** Representative histograms showing CD103 expression in M-CSF differentiated BMDMs treated with DMSO or a p38 MAPK inhibitor (SB202190), either unstimulated or stimulated with Poly (I:C) for 24h. **(B)** PI staining shows comparable cell viability between DMSO and p38 inhibitor treated groups, indicating that SB202190 treatment did not induce cell death. **(C)** Quantification of CD103 expression from **(A)**, shown as percentage of CD103^+^ cells. **(D)** Rt-qPCR analysis of CD103 (ITGAE) mRNA expression under the same conditions. Data points shown are representative of three independent experiments ± SEM, where each experiment included three biological replicates. A one-way ANOVA with Tukey’s multiple comparisons test was used to determine statistical significance, ****p<0.0001.

Because poly(I:C) stimulation promotes CD103 expression in M-CSF-differentiated MΦ, we examined whether p38 MAPK signaling contributes to this process. BMDMs were pretreated with the p38 inhibitor SB202190 prior to poly(I:C) stimulation and analyzed by flow cytometry (**Figure 3A**). Propidium iodide (PI) staining confirmed that cell viability was unaffected by the inhibitor (**Figure 3B**). Baseline CD103 expression was minimal in all conditions, and poly(I:C) treatment significantly increased CD103 in cells exposed to DMSO alone (**Figures 3A–C**). However, inhibiting p38 MAPK abrogated CD103 upregulation, reducing it to baseline levels (**Figures 3A–C**). RT-qPCR analysis confirmed these findings, revealing that p38 MAPK inhibition suppressed CD103 mRNA (**Figure 3D**). We tested another endosomal TLR ligand (R848, TLR7) to determine if p38 MAPK’s role in CD103 induction is observed with other stimuli. Similarly to poly(I:C), R848-induced CD103 upregulation in M-CSF MΦ was diminished by p38 MAPK inhibition (data not shown). These results demonstrate that p38 MAPK signaling plays a crucial role in mediating CD103 expression in M-CSF-differentiated MΦ.

### 3.4 Peritoneal MΦ (pMΦ) express CD103 upon LCMV infection *in vivo*

**Figure 4:**
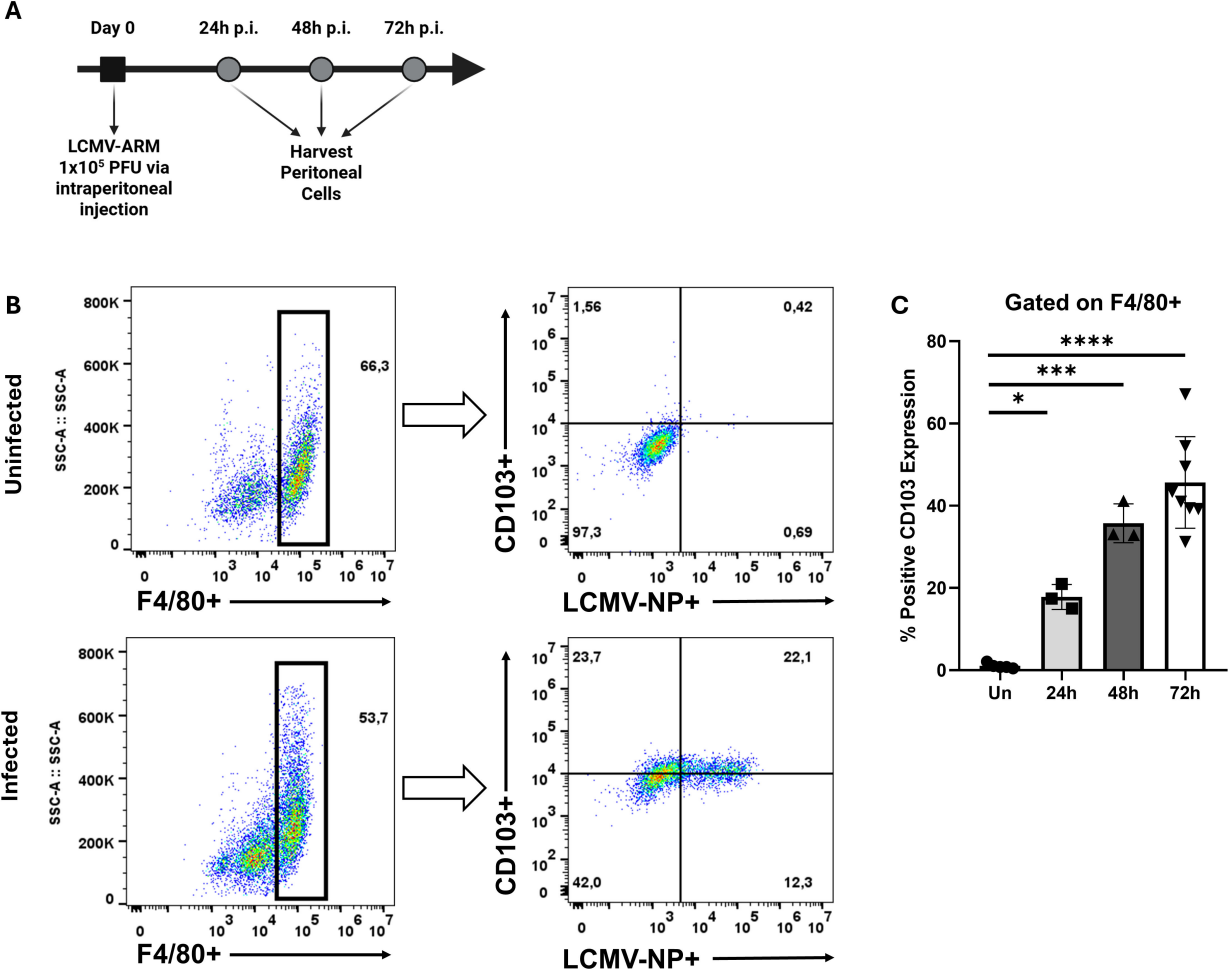
LCMV infection induces CD103 expression on peritoneal macrophages. **(A)** Schematic representation of experimental workflow. **(B)** Representative flow cytometry plots of peritoneal cells harvested at day 3 post-infection with LCMV-ARM (1x10^5^ PFU, i.p.). The left panel shows the gating strategy used to identify F4/80^+^ macrophages, while the right panel displays CD103 expression versus LCMV nucleoprotein (NP) expression within the F4/80^+^ population. **(C)** Quantification of CD103 expression over time following infection, shown as percentage of CD103^+^ macrophages at 24, 48, and 72h post-infection. Each data point represents an individual mouse. A one-way ANOVA with Tukey’s multiple comparisons test was used to determine statistical significance, *p<0.05, ***p<0.001, ****p<0.0001.

To test whether viral infection induces CD103 expression on peritoneal MΦ (pMΦ) *in vivo*, mice were infected intraperitoneally with 1×10^5^ PFU LCMV-ARM. Peritoneal cells were isolated on days 1, 2, and 3 post-infection and analyzed by flow cytometry to track CD103 expression (**Figure 4A**). MΦ were identified by gating on F4/80+ cells (**Figure 4B**), and LCMV-infected cells were detected by staining for the nucleoprotein (NP). At 24 hours post-infection, ~17.8% of pMΦ expressed CD103 (**Figure 4C**). This population expanded over time, reaching ~35.7% at 48 hours and ~45.7% at 72 hours, indicating progressive induction of CD103. Uninfected pMΦ expressed minimal CD103, consistent with previous reports (Hashimoto et al., 2011; Gautier et al., 2012; Bain et al., 2016; Ley et al., 2016). Notably, CD103 was upregulated in both NP+ (infected) and NP- (bystander) pMΦ, suggesting that paracrine inflammatory signals support CD103 induction. These data show that LCMV infection triggers *de novo* CD103 expression in MΦ, challenging the notion that CD103 is restricted to DCs and T cells, and highlight its broader role in immune cell interactions.

## 4 Discussion

CD103 expression has been extensively described in cDC1 and tissue-resident T cells (Ng et al., 2018; Szabo et al., 2019), yet its regulation and function in MΦ remain largely unexplored. Our findings demonstrate that CD103 can be induced in MΦ under specific inflammatory conditions, indicating that local stimuli, rather than lineage-specific factors, govern its expression. This challenges the notion that CD103 is confined to conventional DCs and T cells and instead supports a broader role in modulating immune responses.

We observed that M-CSF-differentiated BMDMs minimally expressed CD103 at baseline but showed robust upregulation after exposure to endosomal TLR ligands (TLR3, TLR7, TLR9) or infection with LCMV. *In vivo*, peritoneal MΦ similarly induced CD103 expression during acute LCMV infection, underscoring the dynamic nature of integrin regulation in response to viral cues. These findings parallel previous observations of β2 integrin upregulation in various immune cells during viral infections, which promotes adhesion, migration, and immune surveillance (Yao et al., 2022). Although CD103 belongs to a distinct integrin family, its induction under viral-driven conditions may reflect a broader phenomenon whereby inflammation and type I interferons (Zhou et al., 2010; Mack et al., 2011) reshape integrin profiles to enhance tissue-specific immune functions.

Interestingly, CD103 induction occurred exclusively in M-CSF-differentiated MΦ, whereas GM-CSF-differentiated MΦ did not exhibit similar responses to inflammatory stimuli. GM-CSF MΦ typically display an M1-like proinflammatory profile (Fleetwood et al., 2007; Lacey et al., 2012a; Alothaimeen et al., 2020; Alothaimeen et al., 2021). This suggests that CD103 expression is not solely a consequence of MΦ activation but is influenced by distinct differentiation programs and signaling pathways. GM-CSF MΦ are known to exhibit enhanced inflammatory cytokine production and increased antigen-presenting capacity (Fleetwood et al., 2007; Lacey et al., 2012a; Alothaimeen et al., 2020; Alothaimeen et al., 2021). Also, GM-CSF MΦ differ in their transcriptional programming compared to M-CSF MΦ (Fleetwood et al., 2007; Lacey et al., 2012a; Lacey et al., 2012b; Zhang et al., 2023), potentially explaining the absence of CD103 induction. Additional studies are warranted to investigate whether other cytokines or transcription factors might override this program, enabling CD103 induction in GM-CSF MΦ under different conditions.

Our mechanistic studies identified p38 MAPK as a key regulator of CD103 expression in MΦ. Inhibition of p38 MAPK strongly suppressed poly(I:C)-driven CD103 upregulation, illustrating how this pathway integrates inflammatory signals to modulate integrin expression. We utilized poly(I:C) due to TLR3 being the only TLR that signals independently from MyD88 (Siednienko et al., 2011), allowing us to delineate overlapping pathways, such as p38 MAPK, which is also engaged by TLR7/9 stimulation (Blasius and Beutler, 2010). Notably, similar p38 MAPK-dependent mechanisms govern CD103 expression in monocyte-derived DCs treated with retinoic acid (Roe et al., 2020). These observations point to convergent signaling pathways, both retinoic acid-dependent and independent, that converge on p38 MAPK to control CD103 induction across multiple immune cell types.

Although endosomal TLR agonists activate p38 MAPK in both M-CSF and GM-CSF MΦ (Alothaimeen et al., 2021), only M-CSF MΦ translate this signalling into CD103 upregulation. Prior work has shown that GM-CSF MΦ mount a stronger pro-inflammatory response upon TLR7 stimulation, characterized by elevated TNF-α and IL-6 production, compared to M-CSF MΦ (Alothaimeen et al., 2021). Interestingly, it has been demonstrated that M-CSF MΦ have higher constitutive IFN-β and basal expression of IFN-stimulated genes than GM-CSF MΦ (Fleetwood et al., 2009). Consequently, in M-CSF MΦ, activation of p38 MAPK and IFNAR dependent pathways potentially synergize to promote CD103 expression, whereas GM-CSF MΦ lacking this IFNAR axis, default to inflammatory cytokine production without CD103 upregulation. *In vivo*, resident pMΦ remain CD103^-^ until LCMV infection provides both TLR7 engagement and elevated levels of type I IFNs (Zhou et al., 2010). However, future studies must explore these mechanistic links to fully elucidate the precise contribution of these pathways to CD103 induction in MΦ.

The functional significance of CD103 expression on MΦ remains to be fully elucidated but is likely tied to interactions with E-cadherin on epithelial cells (Xu et al., 2022). In DCs and T cells, this interaction is vital for retention within epithelial tissues, thereby enhancing local immune surveillance (Ruane and Lavelle, 2011; Xu et al., 2022). For MΦ, the upregulation of CD103 could similarly promote their retention in epithelial-rich environments, potentially influencing tissue-specific immune responses. Moreover, the presence of CD103 may indicate certain MΦ activation states thereby modulating immune responses during inflammation and infection. While our data demonstrate that MΦ can upregulate CD103, the functional implications of this integrin on MΦ remain unclear. Future work should address how CD103 influences MΦ function in epithelial environments, whether CD103 MΦ also contribute to inflammation resolution (Bernatchez et al., 2015), and whether its presence correlates with particular MΦ subsets or activation profiles.

In summary, this study revises the paradigm of CD103 expression by revealing its induction in MΦ under defined inflammatory conditions via p38 MAPK signaling. Our data highlight the plasticity of MΦ in response to viral infection (Alothaimeen et al., 2020; Alothaimeen et al., 2021), emphasizing that local inflammatory cues can substantially reshape integrin expression. These insights deepen our understanding of tissue-specific immunity and may lead to novel strategies for modulating MΦ function under pathophysiological conditions.

## Data Availability

The original contributions presented in the study are included in the article/supplementary material, further inquiries can be directed to the corresponding author/s.
